# Analysis of the impact of urological malignant tumor surgery on child–pugh classification and prognosis in patients with liver cirrhosis

**DOI:** 10.3389/fsurg.2025.1716296

**Published:** 2026-01-07

**Authors:** Yujie Wang, Zhen Huang, Yu Zhang, Wenrui Xue

**Affiliations:** 1 Department of Hepatology Division 2, Beijing Ditan Hospital, Capital Medical University, Beijing, China; 2 Beijing Youan Hospital, Capital Medical University, Beijing, China

**Keywords:** child-pugh classification, liver cirrhosis, prognosis, radical surgery, urological malignant tumor surgery

## Abstract

**Objective:**

Patients with liver cirrhosis often present with relative contraindications to surgery, such as impaired coagulation function and thrombocytopenia.

**Methods:**

A total of 69 patients with urinary system malignancies who underwent radical resection under general anesthesia in the urology department of our hospital between 2013 and 2025 were included in this study. A Wilcoxon rank sum test was conducted to evaluate changes in the Child–Pugh(CP) classification before and after general anesthesia in patients with urinary system malignancies complicated by liver cirrhosis. The Pearson chi-square test was employed to assess the impact of various liver cirrhosis-related factors on the likelihood of CP classification downgrade.

**Results:**

A total of 54 patients were classified as CP grade A prior to surgery. For patients with malignant tumors of urology combined with cirrhosis, the mean CP score (*P* < 0.0001) increased before and after surgery. There was no statistically significant effect of various liver cirrhosis-related factors on deterioration in the CP classification (*P* = 0.072). Univariate and multivariate analyses demonstrated that CP classification downgrading was significantly associated with overall survival (OS) (*P* = 0.006), cancer-specific survival (CSS) (*P* = 0.028), and disease-free survival (DFS) (*P* = 0.039). Compared with patients with a downgraded CP classification, patients without a downgraded CP classification had a more favorable survival curve (OS, *P* = 0.0034; CSS, *P* = 0.0194; DFS, *P* = 0.0296).

**Conclusion:**

Surgery for malignant urinary system tumors in patients with CP grades A and B is generally considered safe. However, in some cases, the procedure may result in an elevated CP score and a downgrade in classification.

## Introduction

In recent years, the number of patients with malignant urinary system tumors requiring radical surgical intervention has increased (1, 2). In addition to tumor-related factors, comorbidities have become increasingly important considerations in clinical decision-making. Over the past two decades, the prevalence and clinical burden of malignancies that coexist with liver cirrhosis have shown an increasing trend (3, 4). Furthermore, the epidemiological profile and burden of liver cirrhosis have been evolving because of increasing rates of obesity and alcohol consumption, as well as improved control of hepatitis B and C virus infections (5). According to global data from 2017, 31.5% of male cirrhosis-related deaths were attributed to hepatitis B, 25.5% to hepatitis C, 27.3% to alcohol-related liver disease, 7.7% to nonalcoholic steatohepatitis, and 8.0% to other etiologies (6). Consequently, urologists are becoming increasingly likely to encounter patients with liver cirrhosis who require radical surgical treatment for genitourinary malignancies. In this specific population, liver cirrhosis develops because of various contributing factors, resulting in varying degrees of impairment in coagulation function, serum albumin concentration, total bilirubin concentration, and even platelet count. These changes consequently increase surgical risks and influence postoperative outcomes. In patients with urological malignancies complicated by liver cirrhosis who are candidates for surgical intervention, preoperative evaluation of liver function using a standardized grading system is essential to determine whether the patient's hepatic function and overall health status can tolerate radical tumor resection. Such surgery involves considerations such as general anesthesia, the extent of the procedure, operative duration, and intraoperative blood loss.

The CP scoring system is widely utilized to assess the severity of liver disease on the basis of five key parameters: the presence and degree of hepatic encephalopathy, the extent of ascites, serum albumin concentration, bilirubin levels, and prothrombin time prolongation. Each parameter is assigned a score, and the cumulative total determines the classification—A, B, or C—corresponding to increasing severity. A higher score indicates more advanced liver disease and is associated with increased surgical risk (7). Furthermore, identifying prognostic factors that influence postoperative outcomes in patients undergoing surgery for urological malignancies is a critical focus of clinical evaluation.

Therefore, incorporating the CP score into a preoperative assessment not only aids in evaluating surgical risk but also provides valuable insight into postoperative prognosis. This information is instrumental in guiding clinical decision-making regarding the appropriateness of surgery and determining the optimal timing for the procedure. For patients who present with a poor preoperative CP classification, efforts are directed toward improving liver function through pharmacological and supportive therapies, with the aim of upgrading the CP grade and thereby expanding the possibility of surgical intervention.

## Materials and methods

### Patients

A total of 69 patients with urinary system malignancies who underwent radical resection under general anesthesia in the urology department of our hospital between 2013 and 2025 were included in this study. All patients had a confirmed diagnosis of liver cirrhosis, namely, 44 cases of hepatitis B-related cirrhosis, 12 cases of hepatitis C-related cirrhosis, 9 cases of alcoholic liver disease, 1 case of autoimmune liver disease, 1 case of primary biliary cirrhosis, and 2 cases of cryptogenic cirrhosis. There were 31 cases of renal cell carcinoma, 25 cases of bladder cancer, 8 cases of upper urinary tract urothelial carcinoma (renal pelvis and ureter), 3 cases of prostate cancer, and 2 cases of penile cancer. The included patients had no concurrent hepatocellular carcinoma or had undergone surgical or interventional treatment for liver cancer.

This study was performed in accordance with the Helsinki Declaration and approved by the Ethics Review Committee of the included hospital. During follow-up, patients or next-of-kin were informed of the study in detail, and informed consent was obtained. The project number is YNKTQN20240913.

### Drug treatment plan for liver cirrhosis

For hepatitis B-related liver cirrhosis, the recommended treatment regimen includes entecavir (0.5 mg once daily) or tenofovir (25 mg once daily). For hepatitis C-related liver cirrhosis, antiviral therapy with interferon is indicated. In cases of alcoholic liver cirrhosis, abstinence from alcohol and nutritional support constitute the cornerstone of management. Additionally, adjunctive therapies such as silybin capsules and enteric-coated adenosylmethionine tablets may be prescribed to help alleviate symptoms associated with liver cirrhosis.

### Laboratory testing

The abdominal color Doppler ultrasound, blood biochemical, and coagulation test results of patients within one week before and after surgery were collected to assess indicators related to ascites, total bilirubin, albumin, and prothrombin time prolongation. On the basis of clinical manifestations and physical signs, the severity of hepatic encephalopathy was classified according to the West Haven criteria.

### Follow-up and study endpoints

One year after surgery, the patient returned to the hospital for follow-up every 3 months, and routine blood sampling, chest x-ray, abdominal color ultrasound, urinary CT, and tumor marker analysis were performed to determine the patient's current prognosis and whether the tumor recurred or metastasized. We considered CSS, OS, and DFS as the end points of the study (in months). CSS was deﬁned as the time from the date of surgery to cancer-related death. OS was deﬁned as the time from the date of surgery to the death of the individual from any cause. DFS was deﬁned as the time from the date of surgery to radiologically or histologically conﬁrmed recurrence or metastasis.

### Statistics

A Wilcoxon rank sum test was conducted to evaluate changes in the CP classification before and after general anesthesia in patients with urinary system malignancies complicated by liver cirrhosis. The Pearson chi-square test was employed to assess the impact of different liver cirrhosis-related factors on the likelihood of CP classification downgrade. Univariate and multivariate analyses were performed to investigate the effects of clinical variables—such as age, sex, hypertension status, diabetes status, coronary heart disease status, surgery duration, blood loss status, and CP classification—on overall survival (OS), cancer-specific survival (CSS), and disease-free survival (DFS). Additionally, Kaplan–Meier (K-M) survival curves were constructed to analyze the influence of changes in the CP classification on the survival outcomes of patients with urinary system malignancies. GraphPad Prism Version 9 (GraphPad Software, La Jolla California, USA; http://www.graphpad.com) was used to generate survival curves, and Wilcoxon rank sum tests were performed. Statistical analysis was performed using SPSS version 23 (SPSS Inc., Chicago, IL, USA). Our laboratories used FACSCanto-II flow cytometers (BD Biosciences, San Jose, CA), and standardized EuroFlow SOPs for instrument setup and calibration were used for the instruments, as provided in detail via the EuroFlow website (http://www.EuroFlow.org).

## Results

### Child–pugh scores before and after surgery

The CP score is used to assess the severity of liver disease on the basis of the degree of hepatic encephalopathy, the degree of ascites, serum albumin and bilirubin levels, and prothrombin prolongation time. Each variable has an A scoring system, and the total score is used to assign grades A, B, or C, with grade A being the best and grade C being the worst (Table 1).

**Table 1:** Child-Pugh scoring interpretation.

Measure	1 Point	2 Points	3 Points
Total bilirubin (umol/L)	<34	34–51	>51
Serum albumin (g/L)	>35	28–35	<28
Prothrombin time (seconds)	<4	4–6	>6
Ascites	None	Mild	Moderate/Severe
Hepatic encephalopathy	None	Grade I-II	Grade III-IV

	Class A	Class B	Class C
Total points	5–6	7–9	10–15

**Figure 1:**
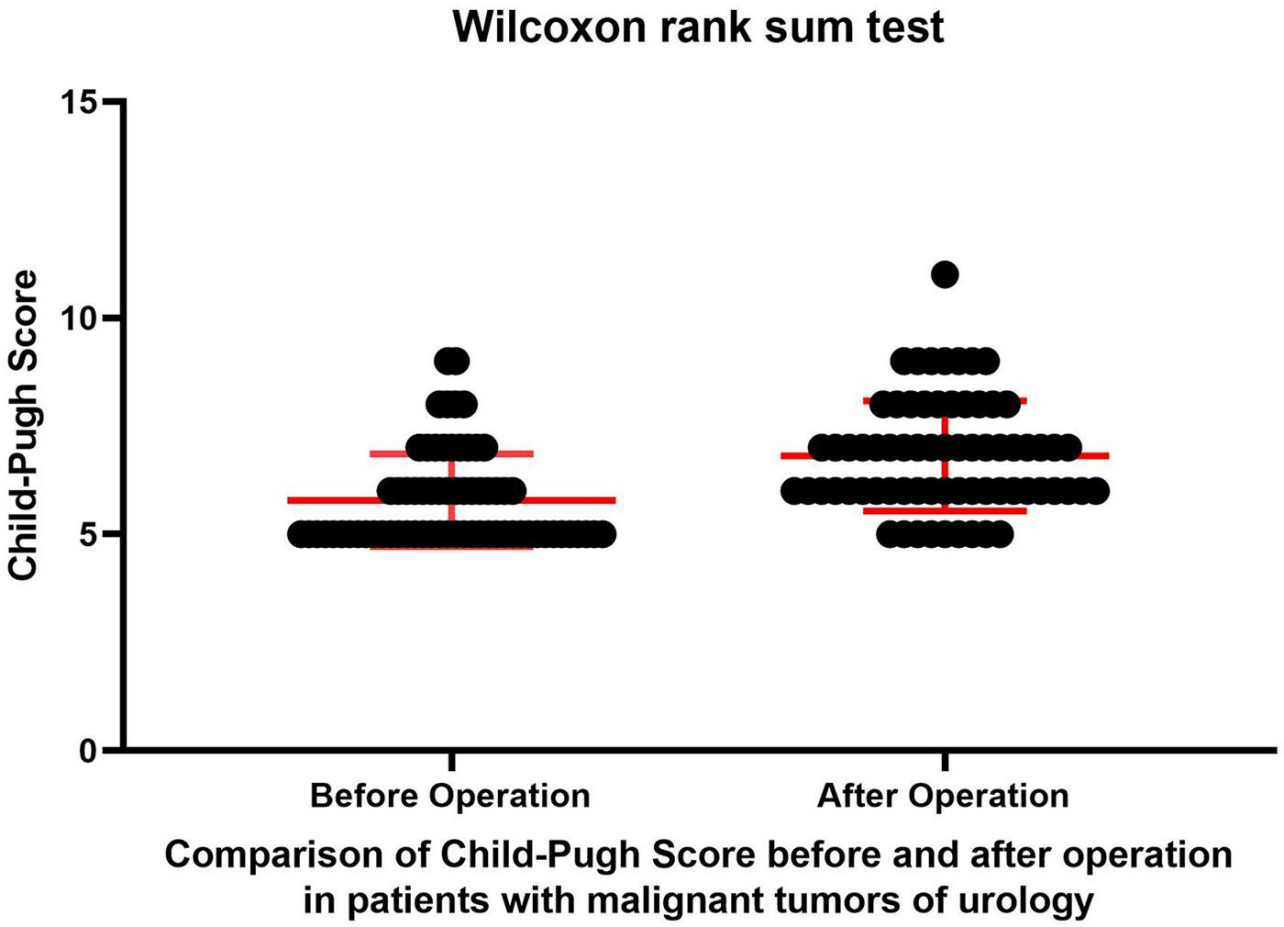
Child–Pugh scores before and after operation.

The results of the Wilcoxon rank sum test revealed that the average CP scores of patients with urinary system malignancies complicated with liver cirrhosis after surgery increased compared with those before surgery (*P* < 0.0001) (Figure 1). As shown in Figure 1, this finding is not related to the type of urinary tract tumor, and the *p*-value is relatively low, which is statistically significant. The narrower confidence interval results were associated with a sufficient number of cases, and the 95% confidence interval in our results did not contain zero, indicating a significant difference. The greater the distance from zero is, the greater the difference. We obtained a value of T = 10.88. The larger the absolute value of T is, the greater the difference in the means of the paired samples. The absolute value of T is greater than 1.96, indicating a significant difference between the paired samples. Except for a few points, most of the points in the graph are located near the average value, which indicates good statistical significance.

### The impact of various etiological factors of liver cirrhosis on the downgrade of the child–pugh classification

**Figure 2:**
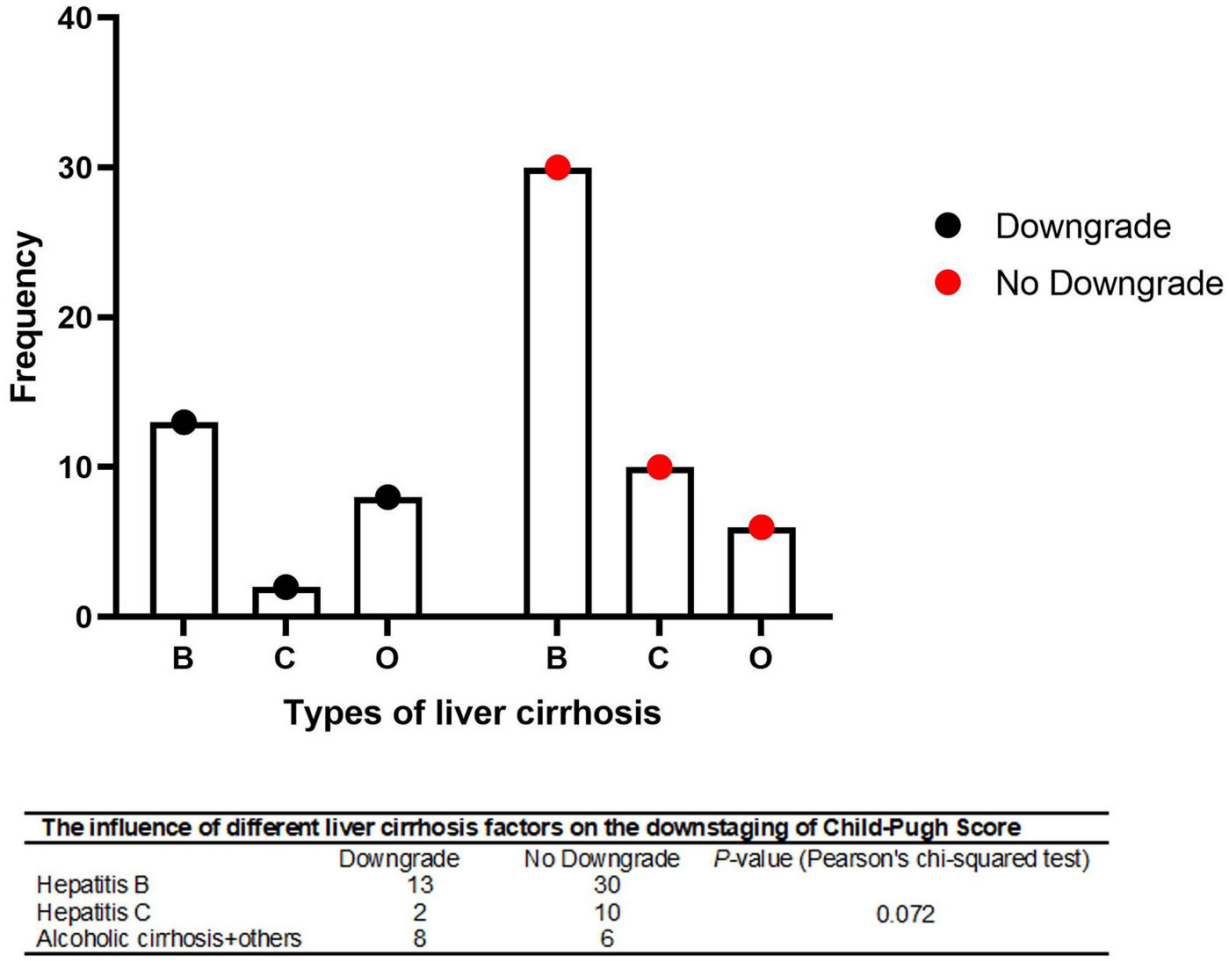
The impact of various etiological factors of liver cirrhosis on the downgrade of the Child–Pugh classification.

As shown in Figure 2, there was no significant difference among the different liver cirrhosis factors (hepatitis B, hepatitis C, alcoholic liver disease and others) in terms of whether the CP of patients with urinary system malignancies complicated with liver cirrhosis was degraded after radical resection (*P* = 0.072). The causes of liver cirrhosis can be roughly classified into viral hepatitis, chronic alcoholic liver disease, nonalcoholic fatty liver disease, long-term cholestasis, drugs or toxins, liver circulatory disorders, genetic and metabolic diseases, immune disorders, or parasitic infections. Liver cirrhosis caused by different etiologies (hepatitis B, hepatitis C, alcoholic liver disease, autoimmune liver disease) is similar in terms of its core pathological changes (destruction of liver structure, formation of pseudolobules, fibrosis), and ultimately, liver function decline and portal hypertension. However, they differ significantly in terms of etiology, pathogenesis, progression rate, risk of comorbidities, treatment focus and some clinical manifestations.

### Clinical variables and the prediction of OS, CSS, and DFS in patients with urological malignancies complicated with liver cirrhosis after radical resection

Among the 69 patients included in the study, the average age was 60 years, and 57 males and 12 females were included. With respect to underlying comorbidities, 15 patients had hypertension, 9 had diabetes, and 5 had coronary heart disease. All 69 patients diagnosed with urinary system malignancies underwent radical surgical treatment, and none received intraoperative blood transfusions. The spectrum of malignant tumors included the most commonly diagnosed urological cancers: renal cancer, bladder cancer, prostate cancer, ureteral cancer, and penile cancer (Table 2). Although the specific types of malignancies varied among patients, no significant differences were observed in baseline characteristics, such as the mean age or length of hospital stay. This homogeneity in clinical baseline data is one of the key reasons for analyzing multiple urinary system malignancies collectively. Additionally, the majority of patients with liver cirrhosis adhered well to medical recommendations, regularly receiving antiviral therapy and abstaining from alcohol consumption.

**Table 2:** Patient characteristics, preoperative and intraoperative data.

Patient characteristics		Renal cancer		Bladder cancer		Prostate cancer		Ureteropelvic cancer		Penile cancer	
Number of Patients		31		25		3		8		2	
Age (years)		58 (46–76)^a^		62 (47–77)^a^		67 (62–69)^a^		59 (40–74)^a^		56 (48–63)^a^	
Gender (M/F)		26/5		22/3		3/0		4/4		2/0	
Hepatitis B cirrhosis		22		12		2		6		2	
Hepatitis C cirrhosis		3		6		1		2		0	
Alcoholic cirrhosis		3		6		0		0		0	
Autoimmune cirrhosis		1		0		0		0		0	
Primary biliary cirrhosis		1		0		0		0		0	
Liver cirrhosis of unknown cause		1		1		0		0		0	
Whether to perform radical surgery		Yes		Yes		Yes		Yes		Yes	
Surgical Method		Radical nephrectomy or partial nephrectomy		Radical cystectomy or partial cystectomy		Radical prostatectomy		Hemiarthroidectomy		Partial penile resection or total penile resection	
Duration of surgery (min)		156 (110–200)		237 (180–300)		250 (200–300)		155 (120–190)		120 (110–130)	
Blood transfusion (mL)		0		0		0		0		0	
Hypertension		6		4		2		2		1	
Diabetes		3		5		1		0		0	
Coronary heart disease		2		1		1		1		0	
Tumor Type		Renal cell carcinoma		Urothelial carcinoma		Adenocarcinoma		Urothelial carcinoma		Squamous cell carcinoma	
Tumor Stage		T1-3N0M0		T2-3N0M0		T1-2N0M0		T1-3N0M0		T1-3N0M0	
Specific liver function indicators (mean value)		Before	After	Before	After	Before	After	Before	After	Before	After
	ALT (U/L)	30.5	28.3	35.2	34.1	15.3	9	22.5	16.6	47.5	22.5
	AST (U/L)	24.5	20.4	27.2	25.6	19.3	16	25.6	18.7	26.5	18.5
	ALP (U/L)	60.5	55.6	50.4	45.2	62.3	48	81.6	64.8	54.5	52.5
	GGT (U/L)	21.5	18.9	23.8	19.7	17	14.3	40.3	27.6	26.5	23.5
Preoperative and postoperative CP scores		A→B	11	A→B	8	A→B	2	A→B	1	A→B	0
		A→A	14	A→A	9	A→A	1	A→A	6	A→A	2
		B→B	6	B→B/ B→C	7/1	B→B	0	B→B	1	B→B	0

ALT, Alanine Aminotransferase; AST, Aspartate Aminotransferase; ALP, Alkaline phosphatase; GGT, Gamma-Glutamyl Transferase; CP, Child–Pugh classification.

a Data is shown as either medians with ranges or number of patients (n) and percentage (%). TNM, Staging methods for malignant tumors of the urinary system.

This study revealed that the male-to-female ratio among patients with liver cirrhosis was 4.75:1. Taking hepatitis B virus (HBV)-induced liver cirrhosis as an example, this disparity may be attributed to hormonal differences between sexes, which influence the progression rates of liver damage and fibrosis caused by chronic hepatitis B. As a result, a greater proportion of males progress to liver cirrhosis (8). Additionally, women generally consume less alcohol and do so less frequently than men do (9), which may also contribute to the observed sex-based difference in the prevalence of liver cirrhosis. Among the patients who were diagnosed with liver cirrhosis in this study, 44 cases were attributed to hepatitis B, 12 to hepatitis C, 9 to alcoholic liver disease, 1 to autoimmune liver disease, 1 to primary biliary cirrhosis, and 2 to unknown etiology. Hepatitis B-related liver cirrhosis was the most common type identified in this study, which aligns with the fact that chronic hepatitis B remains the leading cause of liver cirrhosis across all regions of China. Given the large population base of individuals infected with HBV in China, the prevalence of hepatitis B surface antigen (HBsAg) in the general population ranges from 5% to 6%. In accordance with the classification criteria established by the World Health Organization (WHO), China is currently categorized as a country with a moderate prevalence of hepatitis B virus infection.

The results of univariate and multivariate analyses revealed that CP stage had a statistically significant effect on OS (*P* = 0.006), CSS (*P* = 0.028), and DFS (*P* = 0.039), whereas other clinical indicators, such as age, sex, hypertension status, diabetes status, coronary heart disease status, surgery time, and blood loss, were not significantly different (Table 3).

**Table 3:** Univariate and multivariate analyses of clinicopathological parameters to predict OS, CSS, DFS in patients with urological tumor surgery complicated with liver cirrhosis.

Factor	OS				CSS				DFS			
	Univariate		Multivariate		Univariate		Multivariate		Univariate		Multivariate	
	HR (95% CI)	*P*-value	HR (95% CI)	*P*-value	HR (95% CI)	*P*-value	HR (95% CI)	*P*-value	HR (95% CI)	*P*-value	HR (95% CI)	*P*-value
Age												
≤65	1.296 (0.594–2.828)	0.515	1.234 (0.488–3.125)	0.657	1.523(0.525–4.419)	0.439	2.307(0.674–7.892)	0.183	1.109 (0.467–2.635)	0.814	1.140 (0.431–3.015)	0.792
>65	1.00 (ref)		1.00 (ref)		1.00 (ref)		1.00 (ref)		1.00 (ref)		1.00 (ref)	
Gender												
Female	0.430 (0.202–0.914)	0.128	0.643 (0.199–2.083)	0.462	0.454(0.158–1.310)	0.144	4.328(0.510–36.719)	0.179	0.463 (0.202–1.060)	0.068	0.796 (0.216–2.929)	0.731
Male	1.00 (ref)		1.00 (ref)		1.00 (ref)		1.00 (ref)		1.00 (ref)		1.00 (ref)	
Hypertension status												
No	1.717 (0.722–4.079)	0.221	2.438 (0.922–6.447)	0.172	1.547(0.425–5.635)	0.508	2.936(0.683–12.624)	0.148	1.599 (0.632–4.047)	0.322	2.187 (0.8–5.980)	0.127
Yes	1.00 (ref)		1.00 (ref)		1.00 (ref)		1.00 (ref)		1.00 (ref)		1.00 (ref)	
Diabetes status												
No	0.760 (0.265–2.176)	0.609	0.681 (0.203–2.282)	0.534	1.157(0.328–4.076)	0.820	1.787(0.392–8.148)	0.454	0.705 (0.211–2.352)	0.569	0.765 (0.206–2.838)	0.689
Yes	1.00 (ref)		1.00 (ref)		1.00 (ref)		1.00 (ref)		1.00 (ref)		1.00 (ref)	
Coronary Heart Disease												
No	0.486 (0.066–3.585)	0.479	0.895 (0.103–7.819)	0.920	0.044(0.000–263.296)	0.481	0.000(0.000-X)	0.989	0.595 (0.081–4.391)	0.610	1.097 (0.131–9.202)	0.932
Yes	1.00 (ref)		1.00 (ref)		1.00 (ref)		1.00 (ref)		1.00 (ref)		1.00 (ref)	
Duration of surgery												
≥200min	1.188 (0.558–2.529)	0.656	0.980 (0.406–2.368)	0.965	1.607(0.515–5.010)	0.413	1.432(0.391–5.246)	0.588	1.344 (0.587–3.075)	0.484	1.198 (0.470–3.056)	0.706
<200min	1.00 (ref)		1.00 (ref)		1.00 (ref)		1.00 (ref)		1.00 (ref)		1.00 (ref)	
Amount of bleeding												
≥50mL	0.369 (0.141–0.970)	0.143	0.537 (0.130–2.229)	0.392	0.207(0.066–0.654)	0.007	0.058(0.006–0.574)	0.015	0.350 (0.132–0.927)	0.035	0.455 (0.102–2.028)	0.302
<50mL	1.00 (ref)		1.00 (ref)		1.00 (ref)		1.00 (ref)		1.00 (ref)		1.00 (ref)	
Child-Pugh												
No Down grade	2.679 (1.330–5.397)	0.006	2.671 (1.238–5.760)	0.012	2.974(1.128–7.841)	0.028	2.862(0.970–8.443)	0.057	2.226 (1.043–4.752)	0.039	2.252 (0.990–5.122)	0.053
Down grade	1.00 (ref)		1.00 (ref)		1.00 (ref)		1.00 (ref)		1.00 (ref)		1.00 (ref)	

As shown in Table 3, specific clinical indicators, such as surgery time (≥200 min or <200 min) and intraoperative blood loss (≥50 mL or <50 mL), did not significantly correlate with OS, CSS, and DFS in patients with liver cirrhosis. These findings suggest that malignancy itself has a more pronounced effect on patient survival prognosis. Furthermore, the statistically significant effect of deterioration in the CP classification caused by surgery on OS, CSS, and DFS highlights the importance of optimizing preoperative liver function reserve and increasing relevant parameters to a higher level, which can significantly increase surgical tolerance and improve postoperative outcomes.

### Prognosis for survival

**Figure 3:**
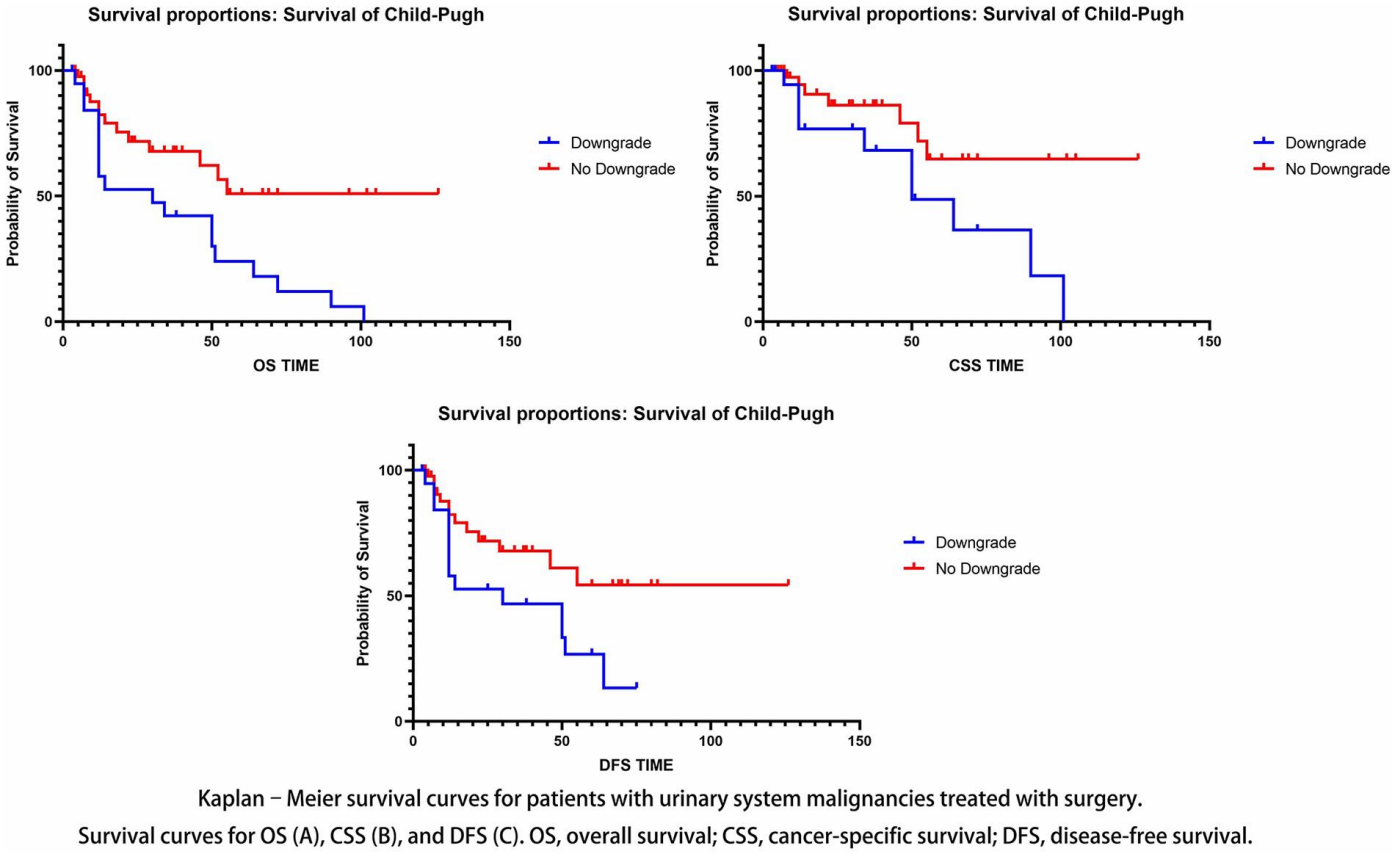
Prognosis for survival.

The survival curve was more favorable for patients with a nondowngraded CP classification than for those with a downgraded CP classification (OS, *P* = 0.0034; CSS, *P* = 0.0194; DFS, *P* = 0.0296) (Figure 3). The mean follow-up duration was 30.5 months, ranging from 3 to 126 months. A decline in CP classification following surgery suggests a general deterioration in the patient's liver function, coagulation capacity, immune resistance, and postoperative recovery ability, thereby indicating a poor prognosis. However, CP classification is not the sole determinant of prognosis. Other factors, such as nutritional indicators, lymphocyte count and serum cholesterol levels, as well as the presence of tumor recurrence and the pathological stage of the tumor, may also significantly influence prognosis. To further validate the statistical significance of the association between CP classification changes and patient survival, the sample size was subsequently expanded.

### The association between perioperative complications and CP degradation

Among 69 patients with urinary system malignancies complicated with liver cirrhosis after radical resection, 2 had bleeding, 10 had fever, 8 had incision infection, 15 had gastrointestinal reactions, 1 had intestinal obstruction, and 5 had costochondral neuralgia. There was no statistically significant difference between postoperative CP degradation and complications. After the model was incorporated into the logistic regression model, the comprehensive test result of the model coefficients was *P* > 0.05, indicating that the model was not significant overall (Table 4).

**Table 4:** Logistic regression analysis of complications after CP downgrading.

Complications	OR (95% CI)	*P*-value
Bleeding		
Yes	NA	*P* = 0.995
No	1.00	
Fever		
Yes	2.503(0.578–10.841)	*P* = 0.220
No	1.00	
Incision infection		
Yes	0.933 (0.175–4.982)	*P* = 0.936
No	1.00	
Gastrointestinal reaction		
Yes	0.394 (0.094–1.648)	*P* = 0.202
No	1.00	
Intestinal obstruction		
Yes	NA	*P* = 0.358
No	1.00	
Costal and abdominal neuralgia		
Yes	1.468 (0.216–9.980)	*P* = 0.694
No	1.00	

### The relationship between platelet counts grouping and CP degradation

The platelet counts were divided into three groups: 0–50, 50–100, and 100–350 × 10^9/L, with 12, 30, and 27 patients included respectively. Through statistical analysis, it was found that there was no statistical significance between platelet counts grouping and whether the postoperative CP grade was downgraded.

## Discussion

The CP classification system incorporates objective laboratory parameters such as serum albumin, bilirubin, and prothrombin time, along with subjective assessments of ascites and hepatic encephalopathy, to stratify patients into three categories—A, B, and C—which reflect varying degrees of hepatic dysfunction (10). This classification is particularly relevant and critical for patients with urinary system malignancies who require surgical intervention, especially when the liver is directly involved or when hepatic function is compromised, as it significantly influences surgical risk and postoperative outcomes. The CP classification serves as a fundamental tool for evaluating hepatic functional reserve, offering a comprehensive assessment of the synthetic, metabolic, detoxification, and excretory capacities of the liver, as well as its ability to withstand surgical stress. Any surgical procedure, particularly major surgery, imposes substantial physiological stress on the body, during which the liver plays a central role in metabolizing anesthetic agents, synthesizing coagulation factors and proteins, eliminating toxins, and modulating immune responses. In patients with impaired hepatic reserve (CP class B or C), the capacity to tolerate surgical stress is markedly diminished, increasing the risk of postoperative hepatic failure—a severe complication associated with a very high mortality rate. Additional risks include coagulopathy (elevating the likelihood of intraoperative and postoperative hemorrhage), hypoalbuminemia (delaying wound healing, exacerbating ascites, and increasing susceptibility to infection), impaired drug metabolism (raising the risk of drug-induced toxicity from anesthetics and analgesics), compromised immune function (increasing the likelihood of postoperative infections), worsening ascites (heightening the risk of wound dehiscence, infection, and herniation), and an increased likelihood of hepatorenal syndrome (11).

### The distinctive characteristics of urological surgery

For patients with urological malignancies requiring surgical intervention, liver function represents a critical determinant in perioperative decision-making. The CP classification serves as a pivotal tool for assessing surgical feasibility, with each grade corresponding to distinct risk profiles. Patients classified as CP A typically present preserved hepatic function and are generally considered suitable candidates for surgery. Patients in grade B require thorough multidisciplinary evaluation because of moderate impairment, whereas patients in grade C are often deemed unsuitable for elective procedures owing to prohibitively high perioperative risks. A systematic assessment of surgical candidacy based on CP stratification is therefore essential (12). In the context of radical surgeries for genitourinary cancers, specific procedural hazards further complicate risk assessment. For example, radical nephrectomy may involve the management of tumor thrombi within the inferior vena cava, and radical prostatectomy is associated with a significant risk of intraoperative hemorrhage—both of which pose substantial threats to patients with underlying cirrhosis. Although these procedures do not directly involve hepatic resection, hemodynamic instability, blood transfusion requirements, and potential renal injury during surgery can precipitate acute liver decompensation. In cases involving ileal conduit urinary diversion, intestinal anastomoses may become particularly vulnerable under conditions of portal hypertension, representing a latent risk for anastomotic leakage or vascular complications (13). Moreover, impaired liver function adversely affects anesthesia management and postoperative recovery, including delayed drug metabolism, coagulopathy, hypoalbuminemia, and heightened susceptibility to infections. Thus, the CP score not only reflects hepatic reserve but also serves as an indicator of overall physiological status. Postoperative complications such as ascites, sepsis, and hepatic failure are more prevalent among cirrhotic patients, especially those with elevated CP scores. Even minor surgical interventions in CP C individuals may trigger fulminant liver failure, rendering surgery contraindicated in most cases.

Furthermore, individual components of the CP system—such as prothrombin time and serum albumin levels—directly influence intraoperative bleeding risk and wound healing capacity (14). Preoperative optimization is therefore paramount and may include correction of coagulopathy, management of hypoalbuminemia, and control of ascites through medical or interventional means. Of particular note is the potential “masking” effect of exogenous albumin administration, which may artificially increase serum albumin levels and thereby lead to the underestimation of true hepatic dysfunction. This limitation underscores the importance of clinical judgment alongside scoring metrics.

Ultimately, the CP classification enables clinicians to identify key areas requiring preoperative intervention and to evaluate whether the achieved optimization is sufficient to justify proceeding with surgery.

### Complications associated with different child–pugh classifications

A meta-analysis conducted by Liu XY et al. revealed that the CP classification significantly influenced postoperative outcomes in patients who underwent colorectal cancer surgery. Specifically, the incidence of postoperative complications among patients with rectal cancer was significantly greater in the CP class B group than in the class A group (*P* < 0.01) (15). A recent study published in a peer-reviewed journal reported an 18% incidence of postoperative liver failure in patients with CP class B disease who underwent robotic prostatectomy. Overall, the complication rates follow a clear hierarchical pattern: class A < class B < class C, with patients in class C exhibiting markedly elevated risk. Similarly, the risk of acute liver failure or acute exacerbation of chronic liver disease following surgery is substantially increased and represents one of the leading causes of postoperative mortality (16, 17). The poorer the Child-Pugh classification, the more likely it is to cause complications such as infection, bleeding, renal failure, impaired wound healing or dehiscence, anastomotic leakage, and worsening of hepatic encephalopathy (18).

### Application of the child–pugh classification system in surgical decision-making

Patients with CP class A cirrhosis are generally considered to have sufficient hepatic reserve to tolerate a wide range of surgical procedures, for example, radical surgeries for urological malignancies—such as radical nephrectomy, radical cystectomy with urinary diversion, and radical prostatectomy—as well as other major abdominal surgeries, such as hepatectomy, pancreaticoduodenectomy, and radical gastrectomy. Nevertheless, their perioperative risk remains higher than that of individuals without liver disease, and they represent the primary candidate population for liver resection, provided that adequate future liver remnant function is preserved (19). Surgical risks in this group are predominantly associated with tumor characteristics and procedural complexity, whereas liver-specific complications remain relatively low. In contrast, patients with CP class B cirrhosis encounter significantly increased surgical morbidity and mortality, rendering such procedures generally contraindicated or high risk, particularly for major abdominal or hepatic surgeries. Clinical decision-making must be exercised with extreme caution, requiring rigorous evaluation of the necessity and urgency of intervention, careful assessment of the risk–benefit ratio, and consideration of limited, life-saving, or palliative procedures only. Hepatectomy is typically deemed either unfeasible or excessively risky in this cohort (20). The anesthesia risk, bleeding risk and infection risk of patients with Child-Pugh B are also significantly increased accordingly (21). Furthermore, perioperative stressors such as surgical trauma, anesthesia-induced hypotension, and blood transfusions may exacerbate underlying liver dysfunction, precipitating postoperative liver failure, hepatic encephalopathy, or hepatorenal syndrome. Surgery in patients with CP class C cirrhosis is associated with prohibitively high mortality and is generally contraindicated. These patients already present decompensated liver function, and even minor surgical interventions may trigger fatal hepatic decompensation. Elective surgery is almost never indicated; emergency procedures may be considered only as a last resort when no alternative exists, although outcomes remain dismal. In the context of hepatocellular carcinoma (HCC), most patients have concomitant cirrhosis, making the CP classification a cornerstone in guiding therapeutic decisions—such as resection, ablation, transarterial chemoembolization, systemic therapy, immunotherapy, and liver transplantation. Hepatic resection is strictly reserved for patients with CP class A and tumors meeting defined oncologic criteria. For those with early-stage HCC and CP class B/C disease, liver transplantation may represent the only potentially curative option, contingent upon meeting transplant eligibility criteria, although the procedure itself is associated with substantial risk (22, 23).

### Prediction of postoperative mortality

CP classification was significantly positively correlated with postoperative mortality, with higher classification grades associated with poorer prognosis (19). The postoperative mortality rate of patients after general HCV-related surgery has been thoroughly studied and is strongly correlated with the severity of liver disease (24) on the basis of the CP classification or the model of end-stage liver disease (MELD), which is represented by the Model for End-Stage Liver Disease (MELD) score (the MELD score is calculated based on objective laboratory parameters, including serum bilirubin, international normalized ratio (INR), and serum creatinine. Subsequently, the model was refined by Kamath et al., with the incorporation of an etiological weighting factor to enhance its prognostic accuracy), and the postoperative mortality rate of patients with CP grade C disease (25) can reach as high as 82%. Individuals with hepatitis C and significant hepatic impairment or decompensated liver disease (CP grades B and C) face a substantially elevated risk of mortality within two years following elective total joint arthroplasty (TJA) compared with those with well-compensated disease (CP grade A). The CP score has been validated as a predictor of mortality following extrahepatic surgical procedures; specifically, the risk of death after abdominal surgery is approximately 30% for patients with CP grade B disease and reaches 82% for those with grade C disease (26). A 2010 review by Modi et al. indicated that multiple small-scale studies and case reports have investigated the influence of liver cirrhosis on postoperative outcomes, consistently showing that patients with high CP scores have higher 30-day morbidity and mortality rates (18). One-year post-operative mortality rates following cardiac surgery among patients with CP grades A, B, and C were reported to be 27.2%, 66.2%, and 78.9%, respectively (27), which aligns closely with findings observed in elective TJA populations. Recent long-term natural history data from HCV-positive cirrhotic patients treated with direct-acting antiviral agents revealed that compared with the general population (5% [95% CI: 3%–7%]), patients with an all-cause mortality risk comparable to that of the general population (5% [95% CI: 3%–7%]) over a five-year follow-up period after adjusting for age and comorbidities. However, standardized mortality analysis using the Human Mortality Database (28) revealed a markedly increased five-year mortality risk among CP grade B patients relative to the general population (25% [95% CI: 13%–37%]). Furthermore, a study by Chisato Okajima et al. demonstrated significant differences in overall survival and disease-free survival between the CPS5 subgroup and the CPS6 subgroup among CP class A patients who underwent hepatectomy for hepatocellular carcinoma (29). These findings underscore the critical importance of liver function in surgical prognosis. Impaired hepatic function not only increases perioperative risk but also adversely affects long-term survival outcomes. Patients with higher CP grades are more susceptible to tumor recurrence, potentially because of compromised immune function and altered drug metabolism. Additionally, such patients often cannot tolerate full-dose chemotherapy regimens, thereby perpetuating a cycle of suboptimal oncologic management.

### Facilitating preoperative optimization

Patients with urinary system malignancies who are scheduled for radical surgery must have a history of liver cirrhosis screened and undergo CP classification assessment. For patients with CP grade B, if surgery is necessary and considered feasible after strict evaluation, active measures should be taken before surgery to optimize liver function status (such as correcting coagulation disorders, supplementing with albumin, controlling ascites, treating hepatic encephalopathy, nutritional support, etc.) to minimize surgical risks as much as possible (30). Perioperative meticulous management includes precise fluid management, avoiding hypotension, selecting appropriate and fast-metabolizing anesthetic drugs, strict hemostasis, preventive use of somatostatin analogs (if portal hypertension is present), prevention of infection, close monitoring of liver and kidney functions and coagulation function, and active management of complications. Moreover, other assessment methods should be integrated, such as the MELD score, imaging examinations, and quantitative liver function tests, to indicate that the CP classification is important but not the only factor. Moreover, the importance of individualized assessment should be emphasized, as the specific conditions of patients may exceed the classification range (31).

### The impact of various etiological factors on liver cirrhosis and their implications for child–pugh classification scores

When evaluating liver cirrhosis patients with different underlying etiologies, are individuals sharing the same CP score of 7 truly comparable? Although the CP scoring system does not differ on the basis of etiology, the pathophysiological differences associated with distinct causes can influence the presentation of specific clinical indicators. For example, hypoalbuminemia tends to be more pronounced in patients with autoimmune liver disease, whereas coagulopathy is often more severe in those with alcoholic liver cirrhosis. While the CP classification employs uniform scoring criteria and categorical stratification (A/B/C) across various etiologies—such as hepatitis B, hepatitis C, alcohol-related, and autoimmune liver diseases—and maintains consistent assessment dimensions, its application may overlook clinically relevant variations. Indeed, liver cirrhosis arising from different etiologies exhibits divergent patterns in disease progression, complication profiles, laboratory manifestations, and therapeutic responses. These disparities may indirectly affect the composition, temporal evolution, and prognostic interpretation of the CP score. Therefore, in clinical practice, it is essential to consider these nuanced differences in conjunction with etiological factors (32). Patients with alcoholic liver disease, hepatitis B- or C-related cirrhosis, and primary biliary cholangitis have an elevated risk of hepatocellular carcinoma. However, the potential association between these conditions and the risk of extrahepatic malignancies remains unclear (4). Furthermore, treatment strategies vary significantly depending on etiology. In hepatitis B-related cirrhosis, the cornerstone of management is long-term, potent antiviral therapy (e.g., entecavir, tenofovir disoproxil fumarate, or tenofovir alafenamide) aimed at sustained viral suppression to undetectable levels, thereby reducing hepatic inflammation, slowing or reversing fibrosis, and decreasing the risk of hepatocellular carcinoma. For hepatitis C-related cirrhosis, the primary objective is viral eradication, which is typically achievable with direct-acting antiviral agents. Nevertheless, even after virological cure, ongoing monitoring of liver function and hepatocellular carcinoma risk—particularly in cirrhotic patients—remains necessary. In patients with alcoholic liver cirrhosis, lifelong abstinence from alcohol constitutes the fundamental prerequisite for any meaningful therapeutic benefit; without complete cessation, all other interventions are rendered ineffective. Concurrent nutritional support (including supplementation of vitamins and protein) and the management of complications are also critical components of care. For autoimmune liver cirrhosis, immunosuppressive therapy forms the core of treatment, requiring long-term maintenance to prevent relapse. Ursodeoxycholic acid is the established first-line therapy and has been shown to improve biochemical parameters and delay disease progression (33, 34).

## Conclusion

The CP classification serves as a critical tool for assessing the surgical eligibility of patients with urological malignancies complicated by liver cirrhosis, as well as for predicting perioperative risks and long-term prognosis. Surgical intervention in these patients is associated not only with potential deterioration in the CP class but also with reduced long-term overall survival, thereby significantly influencing clinical decision-making. Surgeons, anesthesiologists, and hepatologists/oncologists routinely rely on the CP classification, integrating it with additional assessments—such as the MELD score, imaging studies, quantitative measurements of liver stiffness and function, patient comorbidities, and tumor staging—to formulate individualized treatment strategies that maximize safety and efficacy. Disregarding the CP classification in surgical planning may result in severe postoperative complications and adverse outcomes.

## Data Availability

The raw data supporting the conclusions of this article will be made available by the authors, without undue reservation.
